# Impaired humoral and T cell response to vaccination against SARS-CoV-2 in chronic myeloproliferative neoplasm patients treated with ruxolitinib

**DOI:** 10.1038/s41408-022-00651-3

**Published:** 2022-04-22

**Authors:** Patrick Harrington, Katie J. Doores, Jamie Saunders, Marc de Lord, Chandan Saha, Thomas Lechmere, Hataf Khan, Ho Pui Jeff Lam, Amy O’ Reilly, Claire Woodley, Susan Asirvatham, Richard Dillon, Natalia Curto-Garcia, Jennifer O’ Sullivan, Shahram Kordasti, Kavita Raj, Michael H. Malim, Deepti Radia, Donal McLornan, Claire Harrison, Hugues de Lavallade

**Affiliations:** 1 Department of Clinical Haematology, Guy’s and St Thomas’ NHS Foundation Trust, London, UK; 2 School of Cancer and Pharmaceutical Science, King’s College London, London, UK; 3 Department of Infectious Diseases, School of Immunology & Microbial Sciences, King’s College London, London, UK; 4 Department of Population Health Sciences, Faculty of Life Sciences & Medicine, King’s College London, London, UK; 5 Department of Medicine and Molecular Genetics, King’s College London, London, UK

**Keywords:** Myeloproliferative disease, Infectious diseases

**Dear Editor,**

Inferior outcomes have been observed with SARS-CoV-2 infection in patients with chronic myeloid neoplasms, emphasising the importance of development of robust immunity in this population. We and others have previously shown that a single dose of vaccine induces an immunological response in most patients with chronic myeloid malignancies [1–4]. However, the relatively small size of our initial cohorts limited analysis of certain subgroups, while other publications did not study the T cell response to vaccination, an essential component of vaccine efficacy [5]. We report here the humoral and T cell responses induced by sequential doses of vaccination against SARS-Cov-2 in patients with chronic myeloproliferative neoplasms as well as the early protective effect on infection in these patients.

Antibody testing was performed using ELISA for anti-S and anti-N IgG as well as neutralising antibody analysis as described previously [2]. T cell analysis was performed using the Fluorospot assay (Mabtech, Stockholm) with analysis on the IRIS reader using RAWspot technology, as described (Supplementary Methods, Supplementary Fig. 1a, b). Testing was performed in 61 patients including 24 with chronic myeloid leukaemia (CML), 11 with essential thrombocythaemia (ET), 13 with polycythaemia vera (PV) and 13 with myelofibrosis (MF) (Supplementary Tables 1 and 2). Patient samples were obtained at a median of 6.4 (IQR 4.9–8.4) weeks from a second dose of vaccine and 16.6 (14.7–18.5) weeks from the first dose. BNT162b2 vaccine (Pfizer, BioNTech) was used in 85.2% patients (52/61) while the remaining patients (14.8%, *n* = 9/61) received the ChAdOx-1-S vaccine (Astrazeneca).

At the time of submission 1/55 (1.8%) patients completing a post-vaccination survey had confirmed Covid-19 infection 4.5 months after receiving a second dose. This patient was taking ruxolitinib (20 mg, BD) and received the ChAdOx-1 vaccine, requiring hospitalisation with oxygen support for 12 days.

Serological analysis was performed in 60 patients following two doses of vaccine. Three patients had evidence of prior infection with elevated anti-Nucleocapsid IgG. Those with previous infection had higher anti-S IgG and neutralising antibody levels following vaccination than those without previous infection (EC50 5715 vs 1350, ID50 2035 vs 622, *p* = 0.0002/0.001, Supplementary Fig. 2a, b). There was strong correlation between total anti-S IgG and neutralising antibody levels with r 0.74 (*p* < 0.001, Supplementary Fig. 3).

An Anti-S IgG response after two doses of vaccine was observed in 91.7% (55/60) of patients, increasing from 81.1% observed in 37 patients after a single dose (*p* = 0.2) (Supplementary Fig. 2c). A response rate of 96% (22/23) was observed in CML patients and 89.2% (33/37) in MPN patients. Patients with prior infection were excluded from subsequent anti-S IgG analyses. In CML patients the mean EC50 after a first dose was 275 compared to 1180 after a second dose (*p* = 0.056) (Supplementary Fig. 2e). Patients receiving the BNT162b2 vaccine had significantly higher anti-S IgG EC50 and neutralising antibody ID50 at 1420 and 691 compared with 154 and 198 in those receiving ChAdOx-1 (*p* = 0.05/0.04, Supplementary Fig. 4a, b). In addition, a negative or borderline anti-S IgG response at the limit of detection was observed in only 12.2% (6/49) of those receiving the BNT162b2 vaccine compared with 50% (4/8) of those receiving ChAdOx-1 (*p* = 0.025, Supplementary Fig. 4c).

Patients without seroconversion after 2 doses included three patients taking ruxolitinib, one on hydroxycarbamide and one on nilotinib. Five (8.3%) patients had borderline positive results including two patients taking ruxolitinib and three with CML on TKI therapy (imatinib *n* = 1, nilotinib *n* = 1, ponatinib *n* = 1). 50% of patients taking ruxolitinib had a negative or borderline response, significantly higher than that observed in patients not taking ruxolitinib and MPN patients not taking ruxolitinib (5/10 vs 5/47, 5/10 vs 1/25, *p* = 0.01/*p* = 0.004, Fig. 1a, b). Mean ruxolitinib dose was higher at 34 mg/day in those with negative or borderline response compared to 23 mg/day in those with a positive response (*p* = 0.14).

**Fig. 1:**
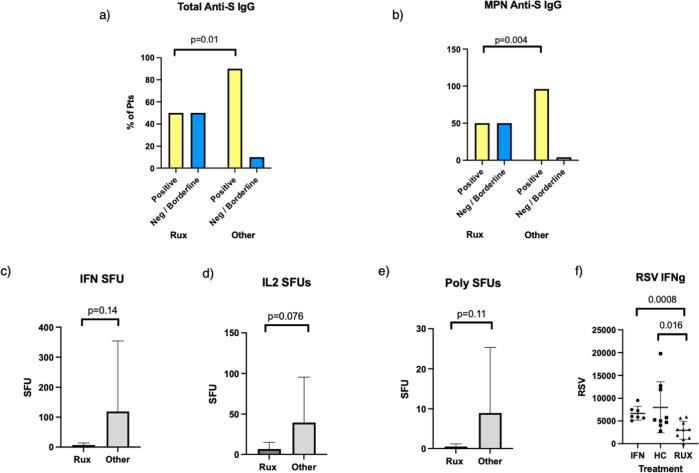
Effect of Ruxolitinib treatment on antibody and T cell response to vaccination. Increased proportion of patients with negative or borderline positive response to vaccination in those taking ruxolitinib compared with (**a**) total cohort of other patients and (**b**) other patients with MPN diagnosis (Fisher’s exact test). **c**–**e** Reduced SFUs for (**a**) IFNg, (**b**) IL-2, and (**c**) polyfunctional cells in patients taking ruxolitinib compared with other patients (Independent samples *t*-test). **f** Reduced RSV in patients on ruxolitinib compared with MPN patients on other therapies (Independent samples *t*-test).

On multivariate analysis (MVA) (Supplementary Methods) for anti-S IgG response there was significant association with ruxolitinib treatment (MV OR = 0.04, 95% CI: 0.00–0.6, *p* = 0.04, Fig. 2) independently of potential confounders including age, sex, treatment, comorbidities, diagnosis and vaccine type. We also observed a significant association between presence of comorbidities and inferior anti-S IgG response to vaccination (MV OR = 0.05, 95% CI: 0.0–0.49, *p* = 0.02, Fig. 2). Vaccine type also showed a trend towards significance with superior anti-S IgG response observed in those receiving BNT162b2 (MV OR = 6, 95% CI: 0.85–48.57, *p* = 0.07, Fig. 2).

**Fig. 2:**
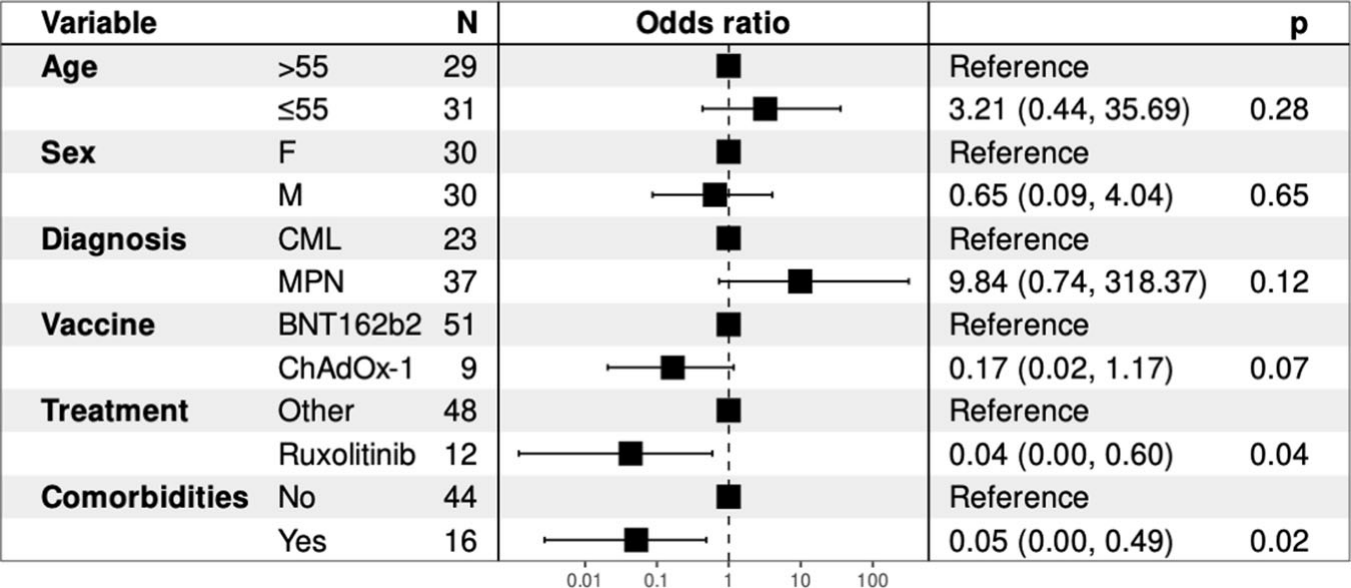
Anti-S IgG Multivariate Analysis. Multivariate analysis of impact of age, sex, diagnosis, vaccine type, ruxolitinib treatment and presence of comorbidities on anti-S IgG response to vaccination.

T-cell analysis was performed in 60 patients after two doses. An overall T-cell response was observed in 88.3% (53/60) patients with an IFNg response observed in 80% (48/60) and an IL-2 response in 68.3% (41/60). In total, participants had a mean of 74 spot forming units (SFU) for IFNg and 26 for IL-2 secretion (*p* = 0.037, Supplementary Fig. 5a). Non-responders included 2 patients taking ruxolitinib, 2 on ponatinib, 1 on hydroxycarbamide and 2 undergoing active surveillance. A polyfunctional T-cell response with secretion of IFNg and IL-2 from the same SFU was observed in 63.3% (38/60) of patients. Excluding previous infection patients receiving ChAd-Ox-1 had lower mean SFUs for IFNg and IL-2 at 9.7 and 3.1 respectively compared with 86.7 and 26.2 in those receiving BNT162b2, although not significant (*p* = 0.2/*p* = 0.2, Supplementary Fig. 4d, e).

Average relative spot volume (RSV) was calculated indicating the amount of cytokine secreted by each cell. Mean RSV of IFNg was higher in reactive polyfunctional T cells at 8665 compared with 5204 within monofunctional cells (*p* = 0.001, Supplementary Fig. 5b). Patients with previous infection were excluded from further analysis of T cell response. Patients with a diagnosis of ET had higher mean SFUs for IFNg compared with other diagnoses with mean of 192.5 in ET, compared with 57.8 in CML, 65.4 in PV and 8 in MF (*p* = 0.075, *p* = 0.2 and *p* = 0.058 respectively, Supplementary Fig. 6a). Similarly mean SFUs for IL-2, was 66.3 for ET compared with 18.3 for CML, 17.5 for PV and 8.7 in MF (*p* = 0.02, *p* = 0.03 and *p* = 0.01 respectively, Supplementary Fig. 6b). Patients with ET had the highest frequency of polyfunctional SFUs with a mean of 14.8 compared with 4.3 in PV patients, 2.9 in CML patients and 0.9 in MF patients (*p* = 0.13, *p* = 0.018, and *p* = 0.037 respectively, Supplementary Fig. 6c).

Patients on ruxolitinib had mean SFUs of 7 for IFNg compared with 119 for MPN patients not taking ruxolitinib (*p* = 0.14, Fig. 1c). Similarly mean SFU for IL-2 was 6.7 for ruxolitinib compared with 39.5 in MPN patients on other treatments or active surveillance (*p* = 0.076, Fig. 1d). Mean polyfunctional SFU was also reduced in those on ruxolitinib compared to other patients with MPN (*p* = 0.11, Fig. 1e). Amongst MPN patients on treatment, RSV of IFNg was lowest in patients on ruxolitinib with a mean average RSV of 2942 compared with 7990 in patients on HC and 6687 in those on pegylated IFNa (*p* = 0.016 and *p* = 0.0008 respectively, Fig. 1f). CML patients taking nilotinib had higher mean frequency of SFUs for IFNg compared to those on other TKI (106 vs 10 *p* = 0.05, Supplementary Fig. 7).

We have previously demonstrated that a single dose of BNT162b2 vaccine elicits both a humoral and cellular response in most patients with chronic myeloid neoplasms [1, 2]. Here we report that a second dose further increases the proportion of patients developing anti-S IgG antibodies and reactive T cells, as well as increasing the anti-S IgG EC50 observed in this patient group. We also demonstrate that patients with previous natural infection have significantly higher anti-S IgG and neutralising antibody levels following 2 doses of vaccine and observed an impaired response in those receiving ChAdOx-1 despite small numbers in this group.

MPN patients are recognised as often having immune deficiency which is heterogeneous and typically associated with a pro-inflammatory state that is influenced by the underlying diagnosis and different treatments. Patients taking ruxolitinib had significantly greater likelihood of having an undetectable or borderline antibody result. In addition, of those taking cytoreductive therapy, patients on pegylated IFNa and HC had significantly greater T-cell RSV for IFNg than patients taking ruxolitinib. We have previously shown that JAK inhibition reduces CD4+ T cell cytokine secretion, which may partially explain the findings observed [6]. In addition, ruxolitinib has been associated with a number of other detrimental effects on the immune system including reduced NK cell and dendritic cell function [7, 8]. Our findings are also supported by those observed in a recent study restricted to humoral responses following a single dose of mRNA vaccine in 18 patients taking ruxolitinib, although conflicting findings have also been reported [9, 10].

CML patients are also recognised to have leukaemia related immune deficiency which is most marked at diagnosis and typically improves with successful TKI therapy [11]. We have previously reported that CML patients, particularly those taking the dual ABL1/SRC kinase inhibitors dasatinib and bosutinib also have potential for significant T-cell dysfunction due to the pivotal role that these kinases play in signalling downstream from the T cell receptor [12]. We found greater IFNg T cell secretion in patients taking nilotinib, which is in keeping with our findings in CD4+ cells after a single vaccine dose [2].

Further studies are required to investigate the degree of antibody and T-cell responses required to provide adequate immunity in these patient groups, as well as longitudinal analysis to assess the dynamics of the response and impact of additional doses.

## Supplementary information

Supplemental Material
