# Supplementary material for: Impaired humoral and T cell response to vaccination against SARS-CoV-2 in chronic myeloproliferative neoplasm patients treated with ruxolitinib
Source: Blood Cancer J. 2022 Apr 22;12(4):73. doi: 10.1038/s41408-022-00651-3 (PMC9024068; doi:10.1038/s41408-022-00651-3)
Supplement: Supplementary file 1 — Supplemental Material [file 41408_2022_651_MOESM1_ESM.docx]

**Impaired humoral and T-cell response to vaccination against SARS-CoV-2 in chronic myeloproliferative neoplasm patients treated with ruxolitinib**

Supplementary Material

Supplementary Methods:

T cell Analysis:

T cell analysis was performed using the Fluorospot assay (Mabtech, Stockholm). Analysis was performed as per manufacturer’s instructions. Briefly, plates were washed with sterile PBS, and then blocked by adding PBS and 10% FCS for 30 minutes. Combined peptide pools covering the S1 region including receptor binding domain (RBD) as well as the S2 domain of the SARS-CoV-2 spike protein were then added to wells at a volume of 50ul per well and concentration of 2ug/ml per well. Anti-CD28 was then added to each well at a concentration of 0.1ug/ml. Anti-CD3 was then added to cells from a healthy control as a positive control. A negative control of PBMCs from a healthy volunteer stored prior to December 2019 was used in each plate. Cells were incubated at 37°C, 5% CO2 for 18 hours. Cells were then removed by emptying the plate and washing. A cocktail of the detection antibodies anti-IFN-y mAb 7-B6-1 BAM and anti-IL-2 mAb MT8G10 were then added to each well and incubated for 2 hours at room temperature. The plate was then again washed and the fluorophore conjugates anti-BAM-490 and SA-550 were added to each well and incubated for 1 hour at room temperature. Fluorescence enhancer was then added and left for 15 minutes at room temperature. Plates were analysed using an IRIS Reader (Mabtech, Stockholm) using RAWSpot technology to provide a 3-dimensional volume for each spot forming unit, labelled as relative spot volume (RSV). Analysis was performed in duplicate and mean values taken. A response was considered positive if there was a 3-fold increase in the number of SFUs from background expression observed in the negative control for each cytokine.

Statistical Analysis:

Antibody and T cell SFU and RSV responses are reported as mean values with comparison of means performed using the independent sample t test. Proportional analyses were performed using Fisher’s exact test. Correlation of anti-S IgG and neutralising antibody ID50 from paired samples was performed Spearman’s rank correlation coefficient. P values were 2 sided and values less than .05 were considered statistically significant. Analyses were performed using SPSS version 24 (IBM, Armonk, NY) and Prism v8. Multivariate analysis was performed with continuous variables described using a summary of central value and dispersion (median and interquartile range) whereas categorical variables were summarized using frequencies. Association between variables were evaluated using Fisher’s exact test or Kruskal-Wallis test as appropriate. Associations between outcome and predictors / confounders was evaluated in the framework of the standard linear model. More precisely, a logistic regression was used for univariate and multivariate regression when the outcome was binary. Odds ratios and regression coefficients are provided along with their 95% confidence interval and significance levels (p values). Computation was realized using the R language for statistical computing version 4.0.3 (2020-10-10).

Supplemental Table 1 – CML Pt Characteristics and immune response post 2^nd^ dose

| **Pt.** | **Age/Sex** | **TKI** | **Dose** | **Anti-N OD** | **Anti S EC50** | **Neut Ab.**  **ID50** | **SFU IFNg** | **SFU IL-2** | **SFU Poly** |
| --- | --- | --- | --- | --- | --- | --- | --- | --- | --- |
| CML1 | 40 / F | BOS | 300 | - | + | + | + | - | - |
| CML2 | 38 / F | PON | 15 | - | + | + | + | + | + |
| CML3 | 50 / M | NIL | 800 | - | + | + | + | + | + |
| CML4 | 24 / M | NIL | 600 | - | + | + | + | + | + |
| CML5 | 74 / M | PON | 15 alt. | - | Weak + | + | + | + | + |
| CML6 | 58 / M | NIL | 600 | - | + | + | + | + | + |
| CML7 | 27 / F | DAS | 100 | - | + | + | + | + | - |
| CML8 | 46 / M | PON | 15 | - | + | + | + | + | + |
| CML9 | 75 / M | BOS | 100 | - | Weak + | + | - | + | - |
| CML10 | 55 / F | IMA | 400 | - | + | + | + | + | + |
| CML11 | 37 / M | NIL | 600 | - | + | + | + | - | - |
| CML12 | 38 / M | NIL | 300 | - | + | + | + | - | - |
| CML13 | 36 / M | PON | 30 | - | + | + | ND | ND | ND |
| CML14 | 40 / M | IMA | 400 | - | Weak + | + | + | + | + |
| CML15 | 59 / M | IMA | 400 | - | + | + | + | - | - |
| CML16 | 61 / M | PON | 30 | - | + | + | - | - | - |
| CML17 | 71 / F | DAS | 70 | - | + | + | + | + | + |
| CML18 | 67 / M | IMA | 400 | - | + | + | + | - | - |
| CML19 | 56 / F | NIL | 600 | ND | ND | ND | + | - | + |
| CML20 | 34 / M | DAS | 100 | - | + | + | + | + | + |
| CML21 | 56 / F | PON | 15 | - | + | + | + | - | + |
| CML22 | 48 / F | NIL | 300 | - | - | - | + | + | + |
| CML23 | 78 / M | PON | 15 | - | + | + | + | + | + |
| CML24 | 57 / M | DAS | 100 | + | + | + | + | + | + |

TKI – tyrosine kinase inhibitor, OD – optical density, EC50 - 50% effective concentration; ID50 WT - 50% inhibitory dilution,

SFU – spot forming units, BOS – bosutinib, PON – ponatinib, NIL- nilotinib, DAS – dasatinib, IMA – imatinib, ND – not done

Supplemental Table 2 – MPN Pt Characteristics and immune response post 2^nd^ dose

|  | **Age/Sex** | **Diagnosis** | **Treatment** | **Anti-N OD** | **Anti S EC50** | **Neut Ab.**  **ID50** | **SFU IFNg** | **SFU IL-2** | **SFU Poly** |
| --- | --- | --- | --- | --- | --- | --- | --- | --- | --- |
| MPN1 | 55 / F | ET | IFN | - | + | + | + | + | + |
| MPN2 | 63 / M | ET | IFN | - | + | + | + | + | + |
| MPN3 | 48 / M | MF | WW | - | + | + | - | - | - |
| MPN4 | 51 / F | PV | WW | - | + | + | + | + | + |
| MPN5 | 44 / F | MF | RUX | - | Weak + | - | + | + | + |
| MPN6 | 64 / F | MF | RUX | + | + | + | + | + | - |
| MPN7 | 65 / F | MF | WW | - | + | + | + | + | + |
| MPN8 | 65 / F | ET | HC | - | + | + | + | + | + |
| MPN9 | 70 / F | PV | RUX | - | + | + | + | + | + |
| MPN10 | 60 / M | ET | IFN | - | + | + | + | - | - |
| MPN11 | 59 / M | MF | RUX | + | + | + | + | + | + |
| MPN12 | 42 / F | ET | WW | - | + | + | + | + | + |
| MPN13 | 58 / F | ET | HC | - | + | + | + | + | + |
| MPN14 | 41 / F | ET | IFN | - | + | + | + | + | + |
| MPN15 | 52 / M | PV | WW | - | + | + | - | - | - |
| MPN16 | 70 / F | PV | HC | - | + | + | + | + | + |
| MPN17 | 76 / F | MF | RUX | - | + | + | - | - | - |
| MPN18 | 43 / M | ET | WW | - | + | + | + | + | + |
| MPN19 | 68 / F | PV | IFN | - | + | + | + | + | + |
| MPN20 | 62 / F | ET | HC | - | + | + | + | + | + |
| MPN21 | 40 / F | MF | WW | - | + | + | + | + | + |
| MPN22 | 51 / F | PV | IFN | - | + | + | + | + | + |
| MPN23 | 60 / F | PV | WW | - | + | + | + | + | + |
| MPN24 | 67 / M | MF | RUX | - | - | - | - | + | - |
| MPN25 | 72 / M | ET | RUX | - | + | + | + | + | + |
| MPN26 | 61 / M | MF | RUX | - | - | 0 | + | + | + |
| MPN27 | 57 / F | PV | HC | - | + | + | + | + | + |
| MPN28 | 66 / F | ET | HC | - | + | + | + | - | - |
| MPN29 | 46 / M | MF | RUX | - | + | + | + | - | - |
| MPN30 | 55 / F | PV | HC | - | + | + | + | - | - |
| MPN31 | 43 / F | MF | IFN | - | + | + | + | + | - |
| MPN32 | 60 / F | PV | HC | - | + | + | - | - | + |
| MPN33 | 56 / M | MF | WW | - | + | + | + | + | + |
| MPN34 | 52 / M | PV | RUX | - | + | + | - | - | - |
| MPN35 | 69 / F | PV | HC | - | - | + | + | + | - |
| MPN36 | 55 / M | MF | RUX | - | - | - | + | - | - |
| MPN37 | 48 / M | PV | RUX | - | Weak + | + | + | - | - |

ET – essential thrombocythaemia, MF – myelofibrosis, PV – polycythaemia vera, IFN – pegylated interferon alpha,

WW – watchful waiting /active surveillance, HC – hydroxycarbamide, RUX - ruxolitin

Supplemental Figure 1


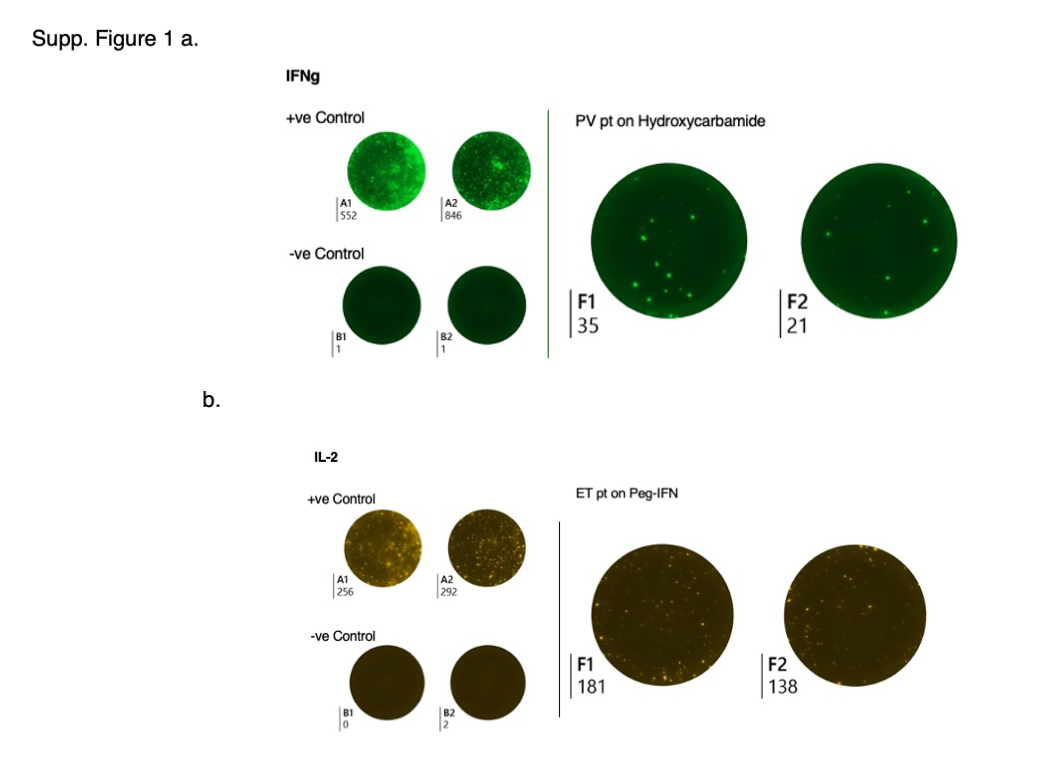


Representative wells analysed in duplicate, showing SFUs for (a) IFNg in patient on hydroxycarbamide (top right) and (b) IL-2 in patient on pegylated interferon (bottom right). Positive (top left) and negative controls (bottom left) for both (a) IFN and (b) IL-2 shown.

Supplemental Figure 2.


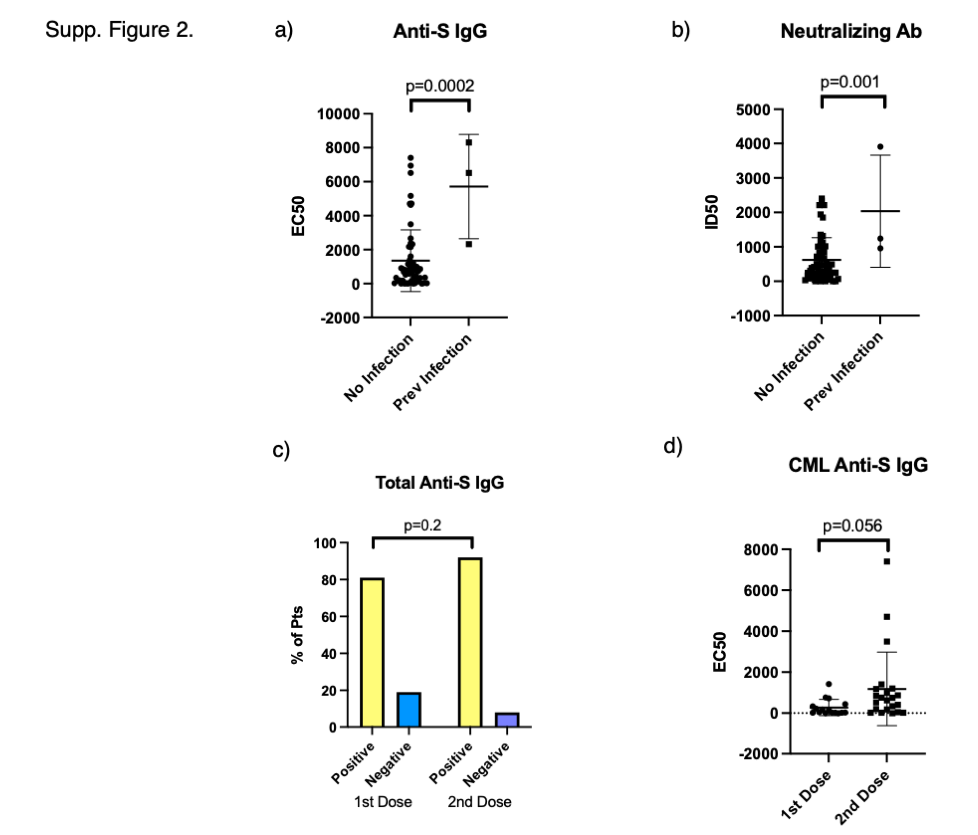


Effect of previous infection and comparison of first and second dose antibody response

a, b, - Increased (a) anti-S IgG EC50 and (b) neutralizing antibody ID50 in patients with evidence of previous infection (Independent samples t-test).

c. Increased proportion of total patients with a positive response after second dose of vaccine although not significant (Fisher’s exact test).

d. Increased anti-S IgG EC50 in CML patients after second dose compared with response to first dose (Independent samples t-test).

Supplemental Figure 3.


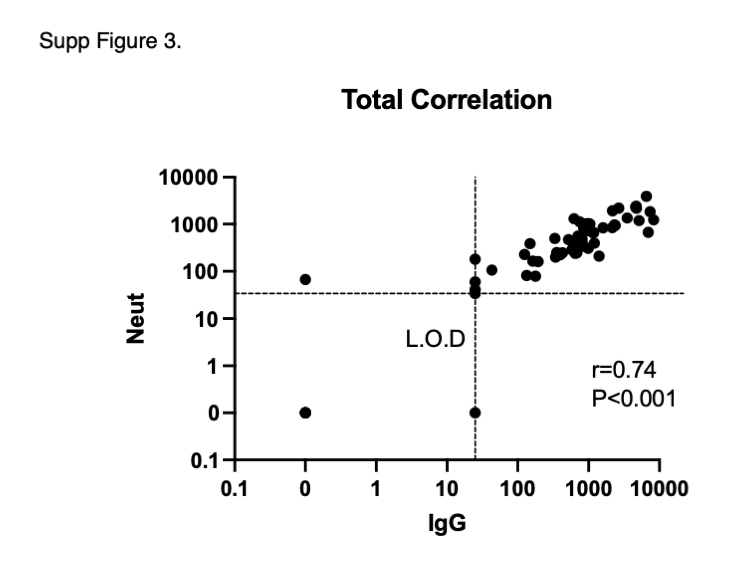


Correlation between anti-S IgG EC50 and neutralizing antibody levels in paired samples across total patient cohort (Pearson correlation coefficient).

Supplemental Figure 4.


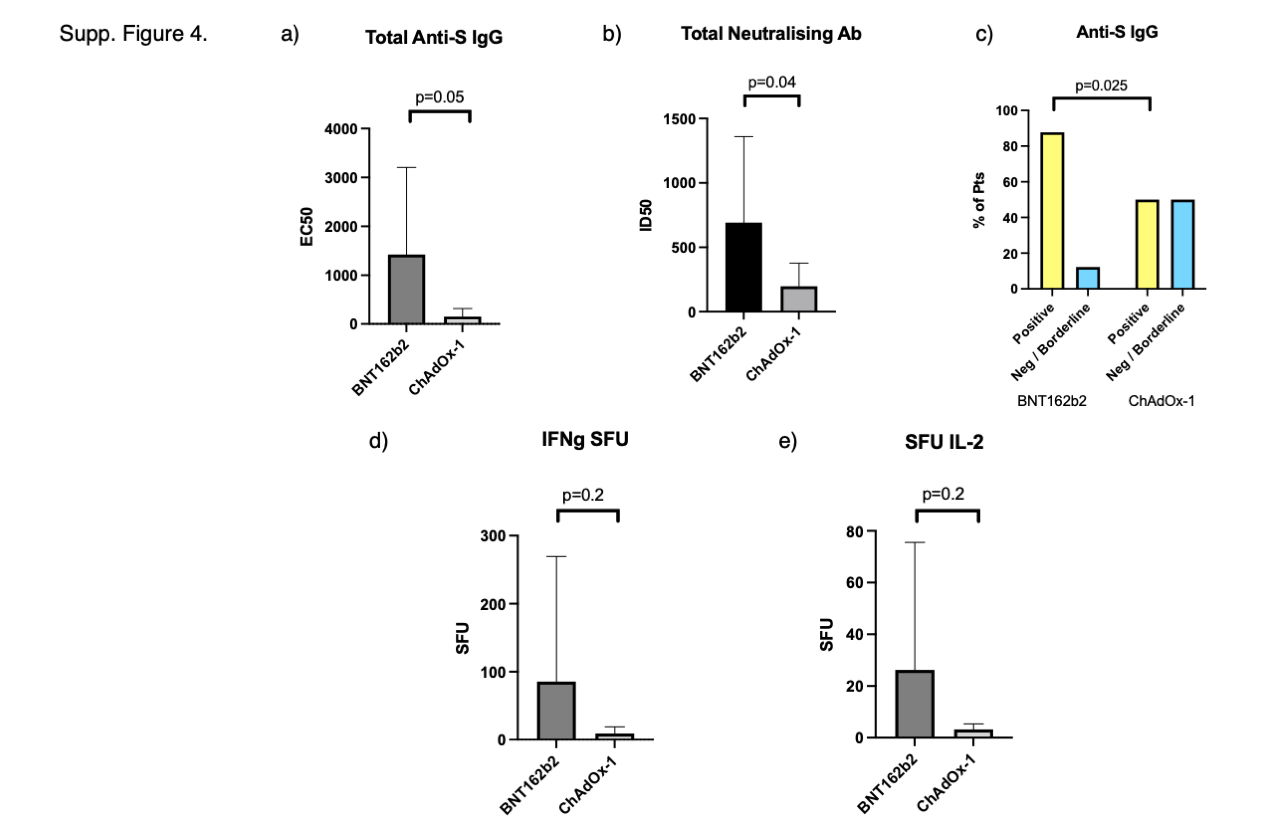


Comparison of response observed in patients receiving different vaccines

a, b, - Increased (a) anti-S IgG EC50 and (b) neutralizing antibody ID50 in patients receiving BNT162b2 compared with ChAdOx-1 vaccine (Independent samples t-test).

c. Increased proportion of patients with negative or borderline positive response to vaccination in those receiving BNT162b2 compared with ChAdOx-1 vaccine (Fisher’s exact test).

d, e, - Increased SFUs for (a) IFNg and (b) IL-2 in patients receiving BNT162b2 compared with ChAdOx-1 vaccine although not significant (Independent samples t-test).

Supplemental Figure 5.


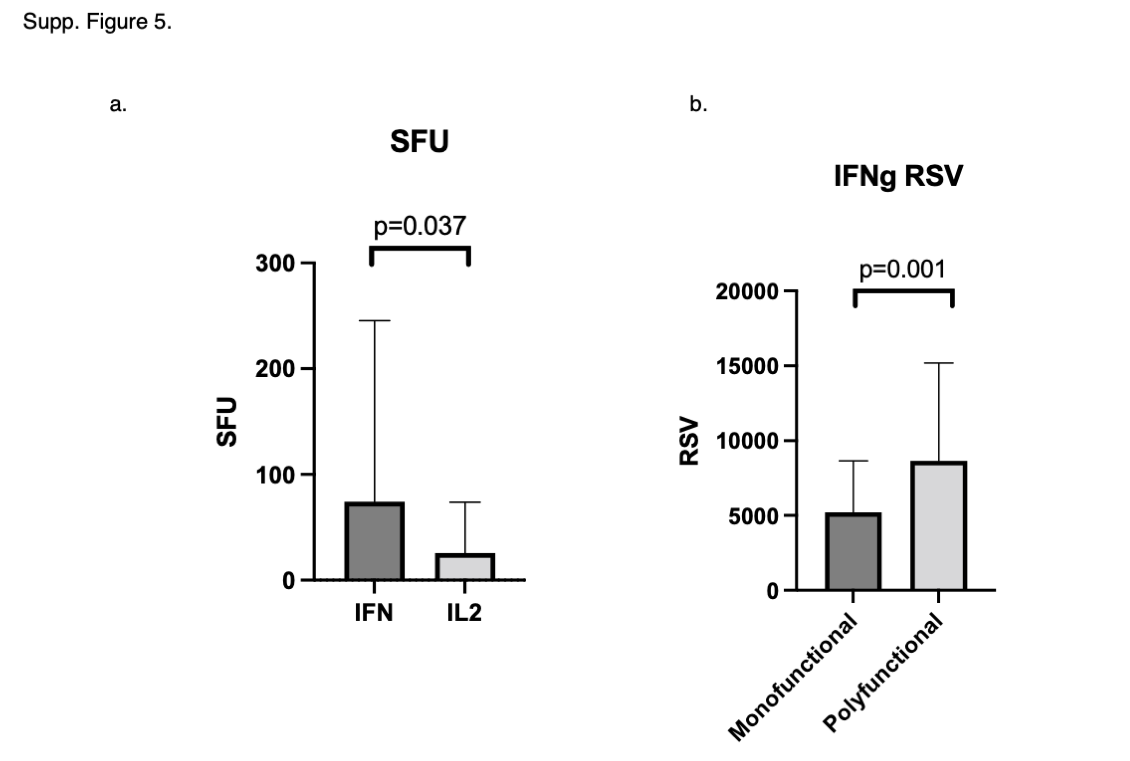


Comparison of mono- and polyfunctional T cell cytokine secretion

a. Increased SFUs for IFNg compared with IL-2 in total patient cohort (Independent samples t-test).

b. Increased IFNg secretion from polyfunctional cells cf. monofunctional cells (Independent samples t-test).

Supplemental Figure 6.


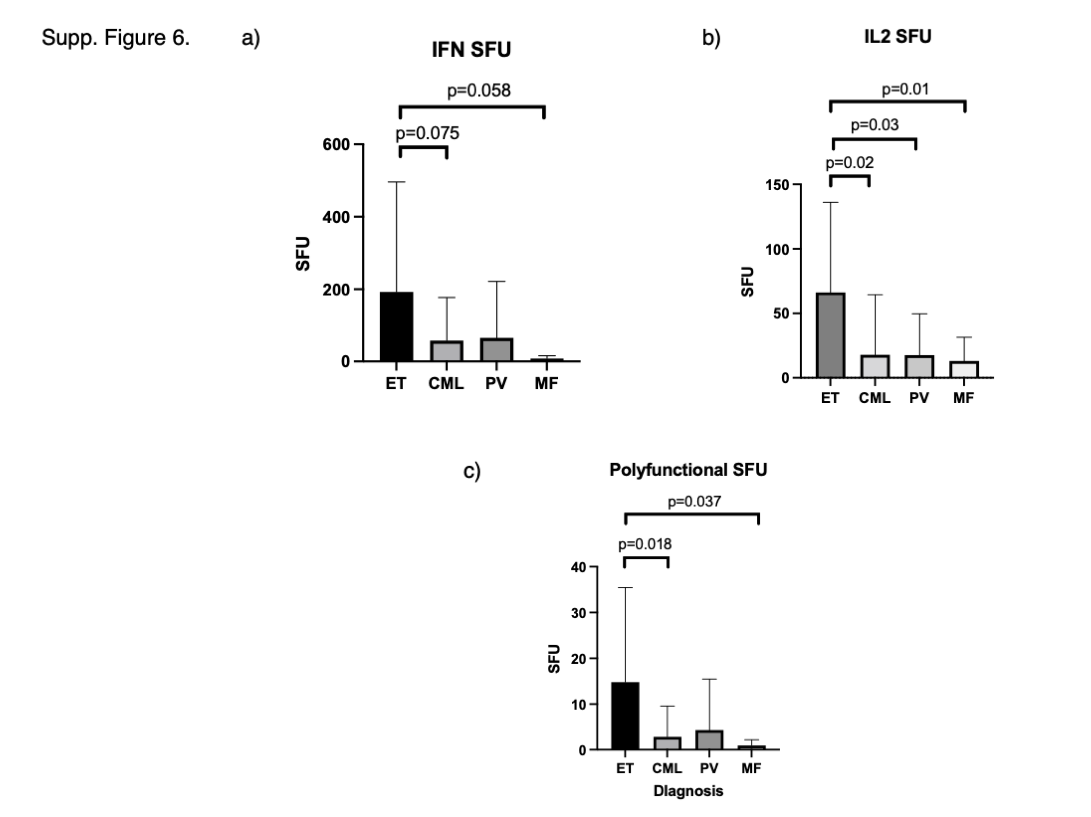


Effect of diagnosis on T cell response

a, b, c. – Increased frequency of SFUs for (a) IFNg, (b), IL-2 and (c) polyfunctional cells in those patients with a diagnosis of ET compared with other diagnostic groups (Independent samples t-test).

Supplemental Figure 7.


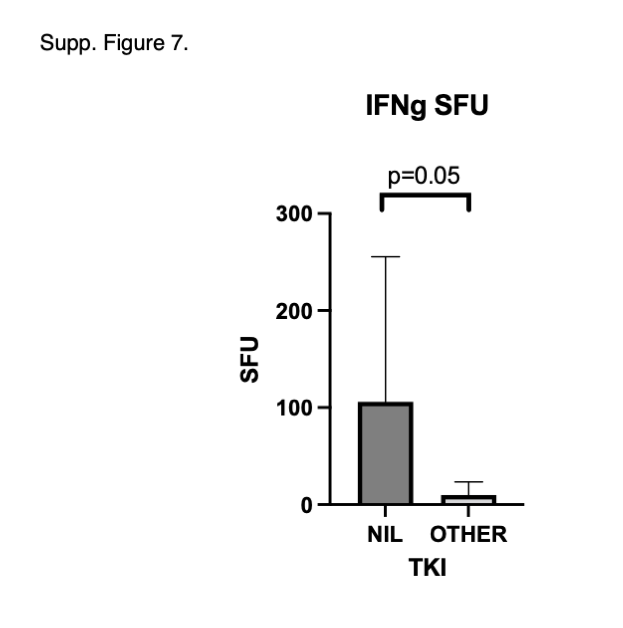


Increased frequency of SFUs for IFNg in CML patients taking nilotinib compared with patients taking other first and second generation TKIs (Independent samples t-test).
